# Development of specific primers for *Fusarium oxysporum* causing damping off of *Lilium formolongi*

**DOI:** 10.1186/s43141-019-0015-2

**Published:** 2020-01-06

**Authors:** Takeshi Toda, Shun Hanesaka, Kuniaki Shishido, Shin-ichi Fuji, Hiromitsu Furuya

**Affiliations:** 1 Department of Bioproduction Science, Faculty of Bioresource Science, Akita Prefectural University, 241-438 Kaidobata-Nishi, Shimoshinjo-Nakano, Akita, 010-0195 Japan; 2 Fukushima Prefecture Office, 2-16 Sugitsuma-cho, Fukushima, 960-8670 Japan

**Keywords:** *Lilium formolongi*, Transposable element, Forma specialis specific

## Abstract

Primers specific for the hypothetical forma specialis of *Fusarium oxysporum* were designed to amplify DNA from this pathogenic fungus that infects plants including lilies. The *F. oxysporum* sequence between the transposal elements *han* and *hop* was used for primer design. Three primer pairs designed from this region were confirmed as specific for 24 isolates of *F. oxysporum* pathogenic to lilies, except for one pathogenic isolates as extraordinary*.* No amplification was observed from *F*. *oxysporum* non-pathogenic to lily, from 12 forma specialis, and 14 fungi and oomycetes concerned with Liliaceae plants. We propose that specific primers designed from this region will be useful to detect isolates of *F*. *oxysporum* that are pathogenic to lilies.

## Introduction

*Lilium formolongi*, known as “Shin-Teppo Yuri” in Japanese, is a lily generated from a cross between *L. longiflorum* and *L. formosanum*, and has been grown on an open-field system in Japan. The flowers of this plant are harvested within 1 year after seeding and replanted every 2 years. Recently, stunted stem growth has been frequently observed at the second cropping season in northern Japan. Stunted lilies have yellow or brownish leaves and yellow or brown roots. *Fusarium*-like fungi have been isolated from the roots of lily plants*.* These isolates were identified as *Fusarium oxysporum* based on their morphology, and pathogenicity was confirmed by an inoculation of the seedlings [1].

*Fusarium oxysporum*, including non-pathogenic varieties, exists ubiquitously in soil. Therefore, a prompt detection technique for pathogenic *F. oxysporum* is desirable. The detection of pathogenic fungi using polymerase chain reaction (PCR) is a popular technique for rapid diagnosis. However, it is difficult to design specific primers for *F*. *oxysporum* forma specialis because their ribosomal DNA sequences and housekeeping genes among forma specialis are very similar [2, 3]. Najafiniya and Sharma [4] reported that random amplified polymorphic DNA markers were available for designing the specific primer for *F*. *oxysporum* f. sp. *cucumerinum*. Also, several reports suggested that the region within or next to transposable elements are useful to design forma specialis- or race-specific primers; *impla* in *F*. *oxysporum* f. sp. *dianthi* [5], and the region between *han* and *skippy* in *F*. *oxysporum* f. sp. *fragariae* [6]. In this study, the primer pairs were designed from the regions between transposable elements for the specific detection of *F*. *oxysporum* pathogenic to lilies.

## Materials and methods

Isolates of *F. oxysporum* were obtained from the roots of *Lilium formolongi* grown at Fukushima Prefecture in Japan. Among seven isolates, pathogenicity on lily plants was confirmed for four isolates by Hanesaka et al. [1], named as lily isolates (Table 1). Tester isolates of *F. oxysporum* described below were used to confirm the specificity of designed primers. An isolate of *F. oxysporum* f. sp. *lilii* (CBS 130322) was provided by the Westerdijk Fungal Biodiversity Institute (Utrecht, the Netherlands). One isolate of *F*. *oxysporum* f. sp. *cucumerinum* isolate and two isolates of *F*. *oxysporum* f. sp. *fragariae* were obtained in Japan. *F*. *oxysporum* f. sp. *lycopersici* race 1, f. sp. *lycopersici* race 2, f. sp. *melonis*, f. sp. *tulipae*, f. sp. *gladioli*, f. sp. *asparagi*, f. sp. *radicis-lycopersici*, f. sp. *raphani*, f. sp. *spinaciae*, f. sp. *dianthi*, and f. sp. *rapae* were provided by the Genebank Project of the National Agriculture and Food Research Organization in Tsukuba, Japan (Table 2). Additionally, 14 isolates of soilborne fungi and oomycetes, reported as pathogenic to lily or possibly concerned with Liliaceae plants, were used for confirming the primers’ specificity (Tables 1 and 2).

**Table 1:** Isolates of *Fusarium oxysporum* obtained from lily used in this study and amplifications by different primer pairs

Isolate no.	Pathogenicity to lily	Host plants	Origin	Designed primer pairs					
				F1/R1^2)^	F1/R2	F2/R1	F2/R2	F3/R1	F3/R2
A1–4	+ ^1)^	Lily	Fukushima, Japan	+ ^3)^	+	+	±	+	+
A2–4	+	Lily	Fukushima, Japan	+	+	+	±	+	+
A2–5	+	Lily	Fukushima, Japan	+	+	+	±	+	+
D2–1	+	Lily	Fukushima, Japan	+	+	+	±	+	+
A2–3	–	Lily	Fukushima, Japan	–	–	–	–	–	–
D1–1	–	Lily	Fukushima, Japan	–	–	–	–	–	–
D2–4	–	Lily	Fukushima, Japan	–	–	–	–	–	–
Ff-3	+	Lily	Fukushima, Japan	±	NT	±	NT	+	NT
Ff-9	+	Lily	Fukushima, Japan	+	NT	+	NT	+	NT
Ff-10	+	Lily	Fukushima, Japan	+	NT	+	NT	+	NT
Ff-13	+	Lily	Fukushima, Japan	+	NT	+	NT	+	NT
Ff-18	+	Lily	Fukushima, Japan	±	NT	±	NT	+	NT
Ff-24	+	Lily	Fukushima, Japan	+	NT	+	NT	+	NT
Ff-31	+	Lily	Fukushima, Japan	±	NT	±	NT	+	NT
Ff-33	+	Lily	Fukushima, Japan	±	NT	±	NT	+	NT
Fc-58	+	Lily	Fukushima, Japan	+	NT	±	NT	+	NT
Fc-59	+	Lily	Fukushima, Japan	+	NT	±	NT	+	NT
Fc-61	+	Lily	Fukushima, Japan	+	NT	±	NT	+	NT
Fc-62	+	Lily	Fukushima, Japan	+	NT	±	NT	+	NT
Fc-65	+	Lily	Fukushima, Japan	+	NT	+	NT	+	NT
Fc-66	+	Lily	Fukushima, Japan	±	NT	±	NT	+	NT
Fc-69	+	Lily	Fukushima, Japan	+	NT	±	NT	+	NT
Fc-70	+	Lily	Fukushima, Japan	+	NT	±	NT	+	NT
Fc-72	+	Lily	Fukushima, Japan	+	NT	±	NT	+	NT
Fc-74	+	Lily	Fukushima, Japan	+	NT	±	NT	+	NT
Fc-77	+	Lily	Fukushima, Japan	+	NT	+	NT	+	NT
Fc-78	+	Lily	Fukushima, Japan	+	NT	+	NT	+	NT
Ff-4	–	Lily	Fukushima, Japan	–	NT	–	NT	–	NT
Ff-4	–	Lily	Fukushima, Japan	–	NT	–	NT	–	NT
Ff-3	–	Lily	Fukushima, Japan	–	NT	–	NT	–	NT
Ff-7	–	Lily	Fukushima, Japan	–	NT	–	NT	–	NT
Ff-8	–	Lily	Fukushima, Japan	–	NT	–	NT	–	NT
Ff-11	–	Lily	Fukushima, Japan	–	NT	–	NT	–	NT
Ff-12	–	Lily	Fukushima, Japan	–	NT	–	NT	–	NT
Ff-16	+	Lily	Fukushima, Japan	–	NT	–	NT	–	NT

^1)^ Pathogenicity to lilies was shown by Hanesaka et al. (2014); +: virulent to lily; −: non-virulent; NT not tested

^2)^ Pairs of Lhs-F1, -F2, -F3, -R1, and -R2 are listed

^3)^ + amplified products were observed, ± faint products were observed, − no amplification was observed

**Table 2:** Isolates of *Fusarium oxysporum*, soilborne fungi, and oomycetes used in this study and amplifications by different primer pairs

Species of isolates	Host plants	Origin	Others	Designed primer pairs					
				F1/R1^2)^	F1/R2	F2/R1	F2/R2	F3/R1	F3/R2
*F. oxysporum* f.sp. *lilii*	Lily	Netherlands	CBS 130322^1)^	+ ^3)^	+	+	–	+	+
*F. oxysporum* f.sp. *gladioli*	Gladiolus	Japan	MAFF305610	–	–	–	–	–	–
*F. oxysporum* f.sp. *tulipae*	Tulip	Japan	MAFF235105	–	–	–	–	–	–
*F. oxysporum* f.sp. *cucumerinum*	Cucumber	Aomori, Japan	–	–	–	–	―	–	–
*F. oxysporum* f.sp. *lycopersici* r.1	Tomato	Japan	MAFF103037	–	–	–	–	–	–
*F. oxysporum* f. sp. *lycopersici* r.2	Tomato	Japan	MAFF238899	–	–	–	–	–	–
*F. oxysporum* f.sp. *melonis*	Melon	Japan	MAFF239211	–	–	–	–	–	–
*F. oxysporum* f.sp. *melonis*	Melon	Japan	MAFF305544	–	–	–	–	–	–
*F. oxysporum* f.sp. *fragariae*	Strawberry	Akita, Japan	No. 1	–	–	–	–	–	–
*F. oxysporum* f.sp. *fragariae*	Strawberry	Akita, Japan	No. 2	–	–	–	–	–	–
*Fusarium oxysporum* f. sp. *radicis-lycopersici*	Tomato	Japan	MAFF103044	–	–	–	–	–	–
*Fusarium oxysporum* f. sp. *raphani*	Radish	Japan	MAFF103057	–	–	–	–	–	–
*Fusarium oxysporum* f. sp. *spinaciae*	Spinach	Japan	MAFF103059	–	–	–	–	–	–
*Fusarium oxysporum* f. sp. *dianthi*	Carnation	Japan	MAFF103072	–	–	–	–	–	–
*Fusarium oxysporum* f. sp. *rapae*	Turnip leaf	Japan	MAFF240321	–	–	–	–	–	–
*Botrytis tulipae*	Tulip	Japan	MAFF245223	–	–	–	–	–	–
*Botrytis* sp.	Korean ginseng	Japan	–	–	–	–	–	–	–
*Rhizopus stolonifer*	*Pandanus* sp.	Japan	MAFF238790	–	–	–	–	–	–
*Cylindrocarpon destructans*	Strawberry	Japan	MAFF306591	–	–	–	–	–	–
*Penicillium* sp.	*Ornithogalum* sp.	Japan	–	–	–	–	–	–	–
*Rhizoctonia solani* AG-4	Unknown	Japan	–	–	–	–	–	–	–
*Sclerotium rolfsii*	Tomato	Japan	–	–	–	–	–	–	–
*Calonectoria ilicicola*	Soybean	Japan	–	–	–	–	–	–	–
*Verticillium dahliae*	Egg plant	Japan	–	–	–	–	–	–	–
*Phytophthora nicotianae*	Chinese chive	Japan	MAFF238148	–	–	–	–	–	–
*Phytophthora cactorum*	Strawberry	USA	MAFF306274	–	–	–	–	–	–
*Phytophthora sojae*	Soybean	Japan	MAFF235802	–	–	–	–	–	–
*Pythium dissotocum*	Rice	Japan	–	–	–	–	–	–	–
*Pythium spinosum*	Cucumber	Japan	–	–	–	–	–	–	–

^1)^CBS: isolate was provided from Westerdijk Fungal Biodiversity Institute (previously Centraalbureau voor Schimmelcultures, the Netherlands). MAFF isolates were provided from the Genebank of the National Agriculture and Food Research Organization (Tsukuba, Japan)

^2)^Pairs of Lhs-F1, -F2, -F3, -R1, and -R2 are listed

^3)^+ amplified products were observed, ± faint products were observed, − no amplification was observed

Isolates were incubated on potato dextrose agar medium at 25 °C for 3 days in the dark, and then transferred onto potato dextrose broth (PDB) for another 3 days. Mycelia were harvested by filtration, washed with sterile distilled water, blotted dry, and transferred into 2 ml tube. Mycelia in the tubes were ground with metal bar by a bubble crasher (Taitech Co., Ltd., Japan). DNA was extracted based on the PEX method [7].

PCR was used to amplify five transposons, *han*, *hop*, *impla*, *hornet*, and *skippy*, using primers hanF/R, hopF/R, implaF/R, hornetF/R, and skippyF/R, and the region between two transposable elements using primer pairs hanFC/RC, hopFC/RC, implaFC/RC, hornetFC/RC, and skippyFC/RC designed by Suga et al. [6]. PCR amplification was carried out using 1 ng of DNA in a 20 μl reaction mixture with 200 μM each of dNTP, 1 μM of the primer pairs, 1× PCR buffer, and 0.5 U AmpliTaq Gold (Applied Biosystems, Foster City, CA). Amplification using the DNA Thermal Cycler (ParkinElmer, Waltham, MA, USA) was done for 1 cycle of 10 min at 95 °C, followed by 35 cycles of 95 °C for 1 min, 55 °C for 1 min, and 72 °C for 3 min, with a final 10 min at 72 °C. Amplification was observed by electrophoresis on 2% agarose gels in Tris-acetate EDTA buffer. The agarose gels were stained with ethidium bromide (10 mg/ml) and visualized under UV transillumination. The success of PCR was confirmed by the presence or absence of fragments from the DNA of *F. oxysporum*.

Amplified products from lily isolates were selected when the size of fragments was distinguished from other isolates, and used for sequencing reactions using a BigDye Terminator kit and a 3700XL Genetic Analyzer (Applied Biosystems). The sequences amplified by these products were used to design the primers, and PCR with designed primers was used to confirm the specificity for lily isolates.

Pathogenicity test of lily seedlings was done using an additional 27 isolates obtained from lily roots (Table 1). The mycelia of 27 isolates grown on PDB were collected and adjusted as 2.5 g. Mycelia were homogenized with 100 ml of sterilized distilled water, mixed with 450 g of soil (Super mix A, Sakata, Japan), and placed into pots. Five lily seedlings grown for 8 weeks (Murakami seed, Ibaragi, Japan) were planted in each pot containing infested soil, and an additional five lily seedlings for control were also planted in non-infested soil. These pots were incubated at 22–27 °C for 4 weeks. When two to five lily seedlings were dead or showed stunting with yellow leaves, isolates inoculated into that pot were considered pathogenic to lilies. When no stunting was observed or only one seedling was stunted, isolates were considered not pathogenic. These isolates were also used for PCR with designed primers.

## Results and discussion

**Fig. 1:**
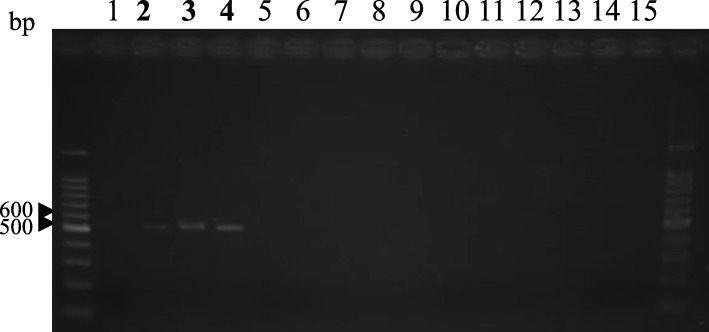
Amplified products obtained by the primer pair hopRC/hanFC. Lanes 1–4: lily isolates of *Fusarium oxysporum* pathogenic to lilies; lanes 5–7: non-pathogenic isolates of *F. oxysporum* obtained from lily roots; lane 8–14: tester isolates of *F. oxysporum*; lane 15: negative control. Marker used was 100 ladder marker

A single 550 bp product was amplified from some lily isolates using primer pair hopRC/hanFC (TTATTCGCACGACCGGTGGTG/ GAACCCTCCAACATTCAACA), and a product with 550 bp was not amplified from non-pathogenic or other isolates of *F. oxysporum* (Fig. 1). DNA sequence of this product from isolate A2–1 was deposited in Genebank (accession no. LC387464). This sequence was not matched with any sequences based on blastn suit search in National Center for Biotechnology Information. Several PCR products by other primers were amplified from lily isolates, but the sizes were not distinguished from other *F. oxysporum*. In addition, there were no useful sequences in five transposons between lily isolates and other tester *F*. *oxysporum*.

**Fig. 2:**
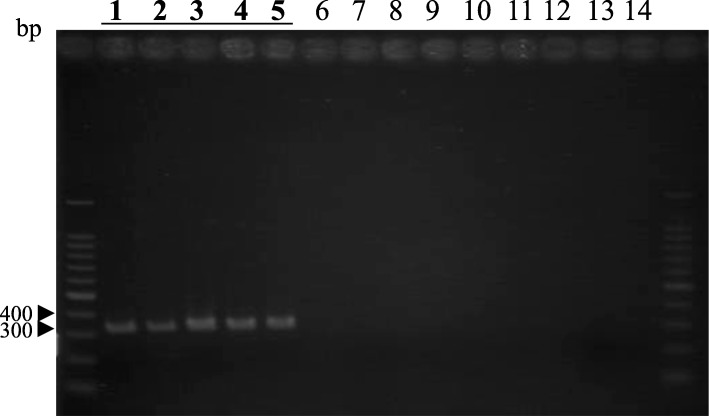
Amplified products obtained by the primer pair Lhs-F1/R1. Lanes 1–4: lily isolates of *Fusarium oxysporum* pathogenic to lilies; lane 5: *F. oxysporum* f. sp. *lilii*; lanes 6–13: tester isolates of *F. oxysporum*; lane 14: negative control. Marker used was 100 ladder marker

Five regions within the sequence amplified by primer pair hopRC/hanFC were used to design the primers. Three regions, Lhs-F1 (TTGAGACTTTGGGGAGGGAGATTT), -F2 (GCTTTGGACTTGAGACTTTGGGGA), and -F3 (CTGCCTTGACTATCTCTAA GCTTT), were used for forward primers, while Lhs-R1 (GTAGCCTACAGCTATCT AT) and -R2 (TCTACCAAATCTATCTACA) were for reverse primers. The primer pairs Lhs-F1/R1, F1/R2, F2/R1, F2/R2, and F3/R1 amplified single products with approximately 350 bp from lily isolates and *F. oxysporum* f. sp. *lilii* (CBS 130322) (Fig. 2 and Table 1), while no products were obtained from other isolates (Tables 1 and 2).

Pathogenicity test resulted that 21 isolates among 28 additional isolates were confirmed as pathogenic, and reisolation was confirmed from the roots of grown lily. Amplification by primer pairs Lhs-F1/R1, F2/R1, and F3/R1 was obtained from the 20 isolates (Table 2). From these, three primer pairs detected about 95% of *F*. *oxysporum* pathogenic to lily. Pathogenic isolate Ef16 would not be lily isolate; however, it was low frequency as less than 5%. Although these primers are still imperfect for preliminary identification of pathogenic *F. oxysporum*, inoculation test indicated that these primer pairs can consistently amplify the fragments of pathogenic isolates. Therefore, these primer pairs are useful for quick diagnosis of this soilborne lily disease or monitoring the pathogens in commercial fields, as it is difficult and time-consuming to identify or specify the pathogens in the soil.

Pathogenicity to lilies has been observed from *F. oxysporum* f. sp. *lilii*, f. sp. *gladioli*, and f. sp. *tulipae* [8]*.* We herein observed amplification by three primer pairs from tester *F. oxysporum* f. sp. *lilii*, but not from f. sp. *gladioli* or f. sp. *tulipae*. Baayen et al. [8] reported that tester isolate CBS130322 of *F. oxysporum* f. sp. *lilii* was not pathogenic in their experiment and that this isolate was different Vegetative Compatibility Group (VCG) from other *F. oxysporum* f. sp. *lilii*. It is possible that this isolate would reduce the pathogenicity to lily by long-time incubation, and that several different VCGs were categorized into the same forma specialis within *F*. *oxysporum* [8]. We therefore suggest that *F. oxysporum* lily isolates obtained from lilies grown in Japan would be close to *F. oxysporum* f. sp. *lilii.*

In summary, because transposable elements exist randomly in the genome of *F. oxysporum*, we considered that several of these or related regions would be unique to forma speciales. Therefore, we designed primers specific for the hypothetical forma specialis of *F. oxysporum* that is pathogenic to lilies following the methodology of [6]. Specific primers were identified in the sequence between the two transposable elements *han* and *hop*, and the specificity of three primer pairs was confirmed using *F. oxysporum* lily isolates causing stunting of lily seedlings (Table 2). These primer combinations will be useful for the direct detection of amplified fragments obtained from pathogenic *F. oxysporum* in lily roots, although future studies should confirm the possibility of quick detection. Additionally, these primers will help to clarify whether isolates of *F. oxysporum* obtained from lily roots or the soil are pathogenic to lilies.

## Data Availability

All data generated or analyzed during this study are included in this published article.

## Abbreviations

*F. oxysporum*: *Fusarium oxysporum*
f. sp.: Forma specialis
PCR: Polymerase chain reaction
DNA: Deoxyribonucleic acid
CBS: Westerdijk Fungal Biodiversity Institute (previously Centraalbureau voor Schimmelcultures, the Netherlands)
MAFF: Isolates provided from the Genebank of the National Agriculture and Food Research Organization (Tsukuba, Japan)
PDB: Potato dextrose broth
VCG: Vegetative compatibility group

## References

[1] Hanesaka S, Shishido K, Toda T, Fuji S, Furuya H (2014) Clarifying factor of replant failure on *Lilium* x *formolongi* in Fukushima prefecture and useful assay of fungal community in roots. Jpn J Phytopatol 80:266 (In Japanese Abstract)

[2] Geiser DM, del Mar Jimenez-Gasco M, Kang S, Makalowska I, Veeraraghavan N, Ward TJ, Zhang N, Kuldau GA, O’Donnell K (2004) FUSARIUM-ID v. 1.0: A DNA sequence database for identifying *Fusarium*. Eur J Plant Pathol 110:473–479

[3] Hirano Y, Arie T (2009) Variation and Phylogeny of Fusarium oxysporum Isolates Based on Nucleotide Sequences of Polygalacturonase Genes. Microbes Environ 24:113–120 10.1264/jsme2.me08554 21566363

[4] Najafiniya M, Sharma P (2011) Specific PCR-based marker for detection of pathogenic groups of *Fusarium oxysporum* f.sp. *cucumerinum* in India. J Genet Eng Biotechnol 9:29–24

[5] Chiocchetti A, Bernardo I, Daboussi MJ, Garibaldi A, Gullino ML, Langin T, Migheli Q (1999) Detection of *Fusarium oxysporum* f. sp. *dianthi* in carnation tissue by PCR amplification of transposon insertions. Phytopathology 89:1169–1175 10.1094/PHYTO.1999.89.12.1169 18944641

[6] Suga H, Hirayama Y, Morishita M, Suzuki T, Kageyama K, Hyakumachi M (2013) Development of PCR primers to identify *Fusarium oxysporum* f. sp. *fragariae*. Plant Dis 97:619–625 10.1094/PDIS-07-12-0663-RE 30722188

[7] Nakahara K, Hataya T, Uyeda I (1999) A simple, rapid method of nucleic acid extraction without tissue homogenization for detecting viroids by hybridization and RT-PCR. J Virol Methods 77:47–58 10.1016/s0166-0934(98)00135-9 10029324

[8] Baayen RP, Forch MG, Waalwijk C, Bonants PJM, Loffler HJM, Roebroeck EJA (1998) Pathogenic, genetic and molecular characterisation of *Fusarium oxysporum* f.sp. *lilii*. Eur J Plant Pathol 104:887–894

